# Co-expression of RON and MET is a prognostic indicator for patients with transitional-cell carcinoma of the bladder

**DOI:** 10.1038/sj.bjc.6602593

**Published:** 2005-05-03

**Authors:** H-L Cheng, H-S Liu, Y-J Lin, H H-W Chen, P-Y Hsu, T-Y Chang, C-L Ho, T-S Tzai, N-H Chow

**Affiliations:** 1 Department of Urology, National Cheng Kung University, 1 Ta-Hsueh Road, Tainan 70428, Taiwan; 2 Departments of Microbiology and Immunology, National Cheng Kung University, 1 Ta-Hsueh Road, Tainan 70428, Taiwan; 3 Department of Radiation Oncology, National Cheng Kung University, 1 Ta-Hsueh Road, Tainan 70428, Taiwan; 4 Institute of Basic Medical Sciences, National Cheng Kung University, 1 Ta-Hsueh Road, Tainan 70428, Taiwan; 5 Department of Parasitology, National Cheng Kung University, 1 Ta-Hsueh Road, Tainan 70428, Taiwan; 6 Department of Pathology, National Cheng Kung University, 1 Ta-Hsueh Road, Tainan 70428, Taiwan

**Keywords:** bladder cancer, protein tyrosine kinases, RON, MET, co-expression, prognosis

## Abstract

Recepteur d'Origine Nantais (RON) is a distinct receptor tyrosine kinase in the *c-met* proto-oncogene family. We examined the mutational and expression patterns of RON in eight human uroepithelial cell lines. Biological effects of RON overexpression on cancer cells were investigated *in vitro*, and the prognostic significance of RON and/or *c-met* protein (MET) expression was analysed in a bladder cancer cohort (*n*=183). There was no evidence of mutation in the kinase domain of RON. Overexpression of RON using an inducible Tet-off system induced increased cell proliferation, motility, and antiapoptosis. Immunohistochemical analysis showed that RON was overexpressed in 60 cases (32.8%) of primary tumours, with 14 (23.3%) showing a high level of expression. Recepteur d'Origine Nantais expression was positively associated with histological grading, larger size, nonpapillary contour, and tumour stage (all *P*<0.01). In addition, MET was overexpressed in 82 cases (44.8%). Co-expressed RON and MET was significantly associated with decreased overall survival (*P*=0.005) or metastasis-free survival (*P*=0.01) in 35 cases (19.1%). Recepteur d'Origine Nantais-associated signalling may play an important role in the progression of human bladder cancer. Evaluation of RON and MET expression status may identify a subset of bladder-cancer patients who require more intensive treatment.

Protein tyrosine kinases (PTKs) are a major class of proto-oncogenes and play a crucial role in many cell regulatory processes, such as proliferation, migration, adhesion, and, potentially, cellular transformation. Currently, most of the established proto-oncogenes in solid tumours are PTKs. The type-1 ErbB family receptors – epidermal growth factor (EGF) receptor and ErbB2 (c-*erb*B-2, HER-2/*neu*) – are well-known examples. Dimerisation by binding two monomers is the regulatory mechanism for the activation of tyrosine kinase receptors (Heldin, 1995). In some cases, formation of heterodimeric complexes allows interaction and cross-talk between different receptors of the same subfamily, and the ErbB receptor family is the best example of homo- and hetero-dimerisation *in vivo* (Wada *et al*, 1990; Sliwkowski *et al*, 1994; Pinkas-Kramarski *et al*, 1996; Chow *et al*, 2001). Therefore, determining the clinical significance of the co-expression pattern of PTKs can provide important molecular targets for cancer therapy.

Recepteur d'Origine Nantais (RON), also known as stem-cell-derived tyrosine kinase in mice, is a distinct receptor tyrosine kinase in the MET proto-oncogene family (Ronsin *et al*, 1993). The ligand for RON was identified as macrophage-stimulating protein (MSP) (Wang *et al*, 1994), expressed by renal tubular cells (Rampino *et al*, 2002). Recepteur d'Origine Nantais induces cell transformation and epithelial tumorigenesis (Collesi *et al*, 1996; Santoro *et al*, 1996; Peace *et al*, 2001; Chen *et al*, 2002). In primary human cancer, highly expressed RON was observed in 47% (35 of 74 cases) of breast cancers and 59.2% (29 of 49 cases) of colorectal cancers (Maggiora *et al*, 1998; Zhou *et al*, 2003).

In our prior profiling experiment (Cheng *et al*, 2002), RON was one of the receptor-type PTKs expressed in bladder cancer cells. Interestingly, this receptor protein and its cognate ligand MSP are all located within chromosome 3p21.3, a region frequently amplified in human bladder cancer (Koo *et al*, 1999). As crosstalk between RON and MET was observed in epithelial cancer (Chen *et al*, 1997; Maggiora *et al*, 2003), we investigated the clinical significance of RON and MET overexpression in human bladder cancer.

## MATERIALS AND METHODS

### Immunoprecipitation

Urine samples (*n*=32) were concentrated using a centrifugal filter device with a molecular weight cutoff of 50 kDa (UltraFree-4; Millipore, Bedford, MA, USA) as described previously (Yan *et al*, 2001). Protein concentrations of the concentrated urine samples were determined using the Micro BCA method (Pierce Chemical, Rockford, IL, USA). Equal amounts of proteins (20 *μ*g) were loaded onto 4–15% gradient gels and separated using sodium dodecyl sulphate (SDS)–polyacrylamide gel electrophoresis under nonreducing conditions. Resolved proteins were electrophoretically transferred to TransBlot nitrocellulose membranes (Bio-Rad, Richmond, CA, USA). The membranes were blocked with 5% low-fat dry milk in TBS-T (10 mM Tris, pH 7.2, 50 mM NaCl, 0.5% Tween 20) for 1 h at room temperature, followed by incubation with primary antibody (1 : 100 dilution) against human MSP*α* (R&D Systems, Minneapolis, MN, USA) at 4°C for 18 h. Blots were washed eight times with TBS-T (5 min per wash) and incubated with a 1 : 5000 dilution of horseradish peroxidase-conjugated secondary antibody (Vector Laboratories, Inc., Burlingame, CA, USA) diluted in TBS-T containing 3% bovine serum albumin for 1 h at room temperature. Labelled proteins were visualised with enhanced chemiluminescence (Amersham Biosciences Corp., Piscataway, NJ, USA). Cell lysates from HepG2 and UB37 were used as positive controls. Informed consent was obtained from all patients before their urine was taken for analysis.

### Cell lines and culture

Four human bladder cancer cell lines (RT4, TSGH-8301, TCC-SUP, and T24) were propagated for use, as described previously (Cheng *et al*, 2002). The locally established cell lines UB09, UB37, UB40, and UB47 were derived from transitional-cell bladder cancer of grade II pT2, grade III pT3, grade III pT1, and grade III pT3, respectively. They were maintained in Dulbecco's modified Eagle's medium (DMEM) (Gibco BRL, Grand Island, NY, USA) supplemented with 10% foetal calf serum (Gibco BRL), L-glutamine (2 mmol l^−1^), sodium pyruvate (110 mg l^−1^), penicillin (100 U ml^−1^), and streptomycin (50 g ml^−1^) at 37°C in a 5%-CO_2_-humidified atmosphere. Primary culture of uroepithelium was obtained from a patient with non-neoplastic urinary disease, and maintained in keratinocyte-SFM (Gibco BRL), supplemented with bovine pituitary extract (20–30 *μ*g ml^−1^), 10% foetal calf serum (Gibco BRL), and EGF (0.1–0.2 ng ml^−1^) as described previously (Adam *et al*, 2003).

### Chemicals and plasmids

Wortmannin was obtained from Sigma (Sigma-Aldrich, St Louis, MO, USA). Plasmid pLP-TRE2-RON was constructed in our laboratory, and co-transfected with pTet-Lac-Hygo in the subsequent experiments as described (Chow *et al*, 2003). In this study, the Tet-off system using doxycycline was used to regulate exogenous RON expression.

### Preparation of total RNA and real-time polymerase chain reaction (RT–PCR) analysis

Total RNA was extracted using Trizol reagent (Invitrogen Corp., Carlsbad, CA, USA). First-strand cDNA was synthesised in a total volume of 20 *μ*l using an M-MLV Reverse Transcriptase kit (Invitrogen). The Roche LightCycler Real-time PCR system (Roche Diagnostics Corporation, Roche Applied Science, Indianapolis, IN, USA) was used to quantify the expression of target genes with SYBR Green I labelling. Polymerase chain reactions were performed in a total volume of 10 *μ*l containing 3 *μ*l cDNA (1 : 100 dilution), 2.5 mM MgCl_2_, 10 pM forward and reverse primers, 1 *μ*l SYBR Green I, and 4.8 *μ*l PCR-grade H_2_O. The reaction was performed for 7 min, to denature DNA and activate the Hot-Start polymerase, followed by 50 PCR cycles denatured at 95°C for 10 s, annealed at 60°C for 5 s, and elongated at 72°C for 12 s in glass capillaries. The primers used were: RON receptor forward 5′-AGCCCACGCTCAGTGTCTAT-3′, RON receptor reverse 5′-GGGCACTAGGATCATCTGTCA-3′; PPIA forward 5′-GTTTGCAGACAAGGTCCCA-3′, and PPIA reverse 5′-ACCCGTAT GCTTTAGGATG-3′.

### Western blot analysis

In all, 50 *μ*g of total protein was denatured in SDS lysis buffer (Tris-HCl (50 mM), pH 6.8, SDS (2%), glycerol (10%), and dithiothreitol (100 mM)) and then loaded into duplicated 12% SDS–polyacrylamide gels. After electrophoresis, one of the gels was stained with 0.5% colorimetric Coomassie brilliant blue (Sigma) as a quantitative control. The other gel was transferred to a polyvinylidene difluoride membrane (Stratagene, La Jolla, CA, USA) and blocked with 5% skimmed milk in PBST (sodium chloride (100 mM), disodium hydrogen phosphate (80 mM), sodium dihydrogen phosphate (20 mM), Tween 20 (0.2%), pH 7.5) solution at 4°C overnight. After being washed with PBST and phosphate-buffered saline, the membrane was hybridised with the monoclonal antibodies for MET, RON, or *β*-actin (Santa Cruz Biotechnology, Inc., Santa Cruz, CA, USA) at 37°C for 1 h. The membrane was then incubated with anti-rabbit or anti-mouse IgG conjugated with horseradish peroxidase at 25°C for 1 h. After being thoroughly washed, the membrane was exposed to radiographic film following a reaction with enhanced chemiluminescence detection reagents (Amersham). Human *β*-actin was used as an internal control.

### Single-strand conformation polymorphism analysis and mutation analysis of the *RON* gene

Each exon of the kinase domain, plus a region containing the proteolytic cleavage site in exon 1, was examined for mutation using a specific PCR primer pair as described previously (Angeloni *et al*, 2000). First-strand cDNAs from primary uroepithelium, UB40, and UB47 cells were prepared using reverse transcription of 1 *μ*g of RNA, according to the instructions for the SuperScript preamplification system (Gibco BRL). The primers used for amplification of RON for PCR were chosen from a prior report (Collesi *et al*, 1996). To check for potential mutations of RON, genomic DNA was also amplified using two primers flanking this region: sense oligomer corresponding to nucleotides 2619–2641 (5′-ATCCACCCAGTGCCAACCTAGTT-3′) and antisense oligomer corresponding to nucleotides 2873–2895 (5′-GGCCAGATGGGGT CCCACA GAG-3′) (Collesi *et al*, 1996). Each sequence was confirmed with two independent PCR reactions.

### Cell viability staining

The cells were re-suspended in DMEM medium. Equal volumes of cell suspension and ethidium-bromide/acridine-orange solution were mixed and placed underneath the cover slides. The cells were observed under a microscope. The live cells labelled with acridine orange are green, and the dead cells labelled with ethidium bromide are orange. The cells were counted and their viability was calculated.

### Wound-healing assay

The UB09 cells (3 × 10^6^) were seeded on a 30-mm dish overnight after transfection. A mid-line wound was made on the monolayer cells, and the healing process was recorded every 20 min until the wound was completely healed (Ho *et al*, 2005). The Image-Pro plus computer program (Media Cybernetics, San Diego, CA, USA) was used to calculate the distance between the wounded edges.

### Clinicopathologic characteristics

For this study, archival blocks were collected from 183 patients (117 men and 66 women; age range: 23–93 years old; mean age: 64.2±10. 4 years) with primary TCC of the urinary bladder treated at our hospital between July 1, 1988, and July 31, 1994. Patients with primary carcinoma *in situ* were excluded from this study. The study protocol was approved by the Human Investigations Committee in our hospital. All cases were reviewed for histological grade according to the World Health Organization classification (Eble *et al*, 2004). Clinical staging was determined according to the Tumor-Node-Metastasis System of the Union Internationale Contre le Cancer (Sobin and Wittekind, 2002) using a survey of the clinical details, image studies, and pathologic data. But no definite tumour staging could be determined at diagnosis in 10 patients (5.5%). This cohort was different from our prior study on MET (Cheng *et al*, 2002).

The treatment and follow-up of patients were conducted according to the standard strategy previously described in detail (Cheng *et al*, 2002). Briefly, all patients with superficial bladder cancer (*n*=93) received transurethral resection and postoperative intravesical chemotherapeutic agent instillation with either thiotepa (30 mg in 30 ml of normal saline for 70 patients) or epirubicin (40 mg in 40 ml of normal saline for 34 patients) weekly for 8 consecutive weeks. Those patients who received intravesical bacillus Calmette-Guerin therapy or neoadjuvant chemotherapy were excluded from this study to preclude any potential bias in prognostic correlation (e.g. recurrence rate or tumour progression). The patients were followed up every 3 months for the first 2 years, every 6 months for another 2 years, and yearly thereafter. Recurrent tumours were confirmed by biopsy, and the patient was given a transurethral operation. This was followed by another 8-week course of intravesical chemotherapeutic instillation therapy or by a radical or partial cystectomy if there was disease progression. Disease progression at recurrence was defined as being at a higher stage than the previous result. In all, 77 patients with muscle-invasive tumours were given a radical cystectomy, and nine patients were given a partial cystectomy. The four patients with distant metastasis at diagnosis received a transurethral resection only. A total of 75 patients received systemic chemotherapy with methotrexate (MTX), cisplatin, doxorubicin, and vinblastine. Survival status was determined by checking outpatient clinic records, interviewing patients' families, or both. Clinical follow-up ranged from 24 to 95 months (median: 54 months).

### Immunohistochemistry (IHC) of RON and MET expression

Immunostaining procedures were described previously (Cheng *et al*, 2002). Briefly, tissue sections were incubated at room temperature for 2 h with monoclonal anti-MET and anti-RON antibodies (Santa Cruz Biotechnology) raised against human MET or RON protein, respectively. The optimal dilution (1 : 100) was determined using human kidney tissue as a positive control (Rampino *et al*, 2002). The StrAviGen Super Sensitive MultiLink kit (BioGenex Laboratories, Inc., San Ramon, CA, USA) was used to detect the resulting immune complex. Peroxidase activity was visualised using an aminoethyl carbazole substrate kit (Zymed Laboratories, Inc., San Francisco, CA, USA). Finally, sections were counterstained with haematoxylin. For the negative control, non-immune mouse immunoglobulin was substituted for the primary antibody in the incubation.

When they evaluated the staining results, the reviewers (N-HC and C-LH) were blinded to the clinical outcomes. Since there was no apparent difference in staining intensity, we used a three-category scoring system for RON and MET based on the proportion of tumour cells stained, as described previously (Chow *et al*, 2001; Cheng *et al*, 2002). ‘High level of expression’ indicates that more than 50% of the tumour cells exhibited immunostaining; ‘low level of expression’, between 10% and 50% reactivity; and ‘negative’, less than 10% or zero staining for RON protein.

### Statistics

Correlations between RON or MET expression and clinicopathologic indicators of bladder cancer, and the biological effects of RON expression on cancer cells were examined, where suitable, using analysis of variance, Fisher's exact test, or *χ*^2^ test. The relationship between IHC expression pattern or biological indicators and clinical outcome was analysed using a multiple logistic regression model. Overall survival was calculated using Kaplan–Meier analysis, and the Cochran–Mantel–Haenszel test (log-rank test) was used to assess the significance of RON and MET expression in relation to tumour recurrence or patient survival. The relative risk (RR) in relation to patient prognosis was assessed using a Cox proportional hazards model after adjustment for clinicopathologic parameters. Only those variables with a *P*-value <0.05 were considered significant.

## RESULTS

### Measurement of MSP in human urine

**Figure 1:**
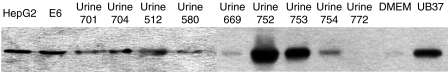
Immunoprecipitation of MSP in human urine. All nine patients with transitional-cell bladder cancer had detectable MSP in their urine, but MSP was not measurable in the urine of patients with xanthogranulomatous pyelonephritis (case 772). The column of DMEM was taken from medium used for culturing of HepG2 and UB37 cells.

To examine the involvement of MSP/RON signalling in bladder carcinogenesis, we analysed the appearance of urinary MSP in a total of 17 cases of urothelial carcinomas (two grade 1, nine grade 2, and six grade 3) and 15 cases of non-neoplastic inflammatory urinary tract diseases. Immunoprecipitation showed that 10 of 17 with cancer and one with an inflammatory urinary tract disease had detectable urinary MSP (*P*=0.04). Representative results of cancer (*n*=9) and xanthogranulomatous pyelonephritis (*n*=1) cases are shown in Figure 1. The appearance of urinary MSP was basically independent of the RON expression status in primary tumours (data not shown). These results imply that the activation of MSP/RON may be involved in the development of human bladder cancer.

### Expression of RON and MET receptors in uroepithelial cell lines

**Figure 2:**
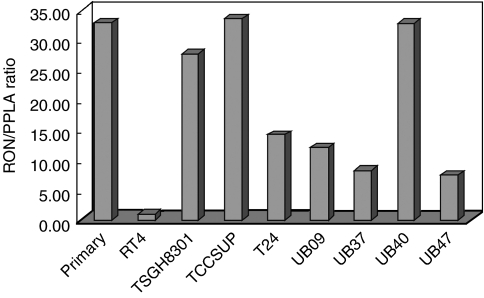
Reverse transcriptase–polymerase chain reaction measurement of RON expression at mRNA level. TCCSUP had the highest level of RON expression in this panel of uroepithelial cell culture, while the other seven cancer cell lines had variable levels of RON expression, with RT4 being the lowest one. Primary culture of uroepithelium was found to have unusually high RON expression. All reactions were performed in triplicate. Data are expressed as relative amounts of RON mRNA levels in relation to PPIA.

The expression of RON was examined at the mRNA level using RT–PCR in a primary culture of uroepithelium and eight cancer cell lines (Figure 2). TCCSUP had the highest level of RON expression in this panel, while the other seven cancer cell lines had variable level of RON expression, with RT4 being the lowest one. Unexpectedly, primary culture of uroepithelium was found to have high RON expression.

**Figure 3:**
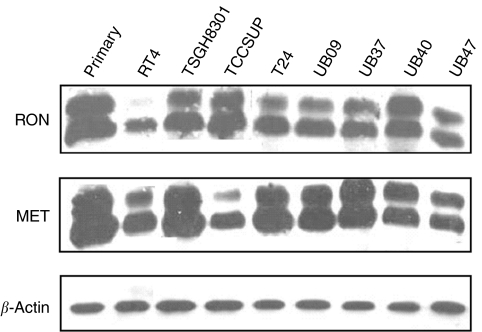
Expression of RON and MET receptor proteins in uroepithelial cells. Primary culture of uroepithelial cells and seven cancer cell lines expressed a mature form of p150^RON^ and a precursor p180^RON^, except that RT cells had very low level of RON expression. All the uroepithelial cells expressed mature p140^MET^ and precursor p170^MET^, except that TCCSUP cells expressed a relatively lower level of precursor p170^MET^. These results indicate that co-expression of RON and MET is a universal event in uroepithelial cells.

Then, expression of RON was assessed at protein level using Western blot (Figure 3). Primary uroepithelial cells and seven of the eight cancer cell lines expressed both the mature form of p150RON and precursor p180RON, except that RT cells had very low level of RON expression. All of the UB series cancer cell lines expressed RON protein. The expression pattern in Western blot basically corresponds to that of RT–PCR. These results suggest that RON-associated signalling events play an important role in the progression of bladder cancer.

However, we have no complete rationale to explain for high level of RON expression in primary culture of uroepithelial cells. A prior study using SV40 large-T antigen immortalised UROtsa cells showed that cells grown in serum-free growth conditions have a phenotype most like the intermediate layers of the urothelium (Rossi *et al*, 2001). Therefore, it is plausible to speculate that primary culture grown in serum-enriched media may exhibit a distinctly different phenotype.

To determine whether constitutive activation of RON is caused by mutations in the kinase domain of RON, genomic DNA was submitted for single-strand conformational polymorphism screening using a panel of intron-based primers covering exon 1 and exons 14–20 of the *RON* gene. The amplified DNA fragments were also sequenced. No mutations were found in the kinase domain of the *RON* gene in any of the cell lines tested (Chow *et al*, 2003).

All of the uroepithelial cells expressed mature p140^MET^ and precursor p170^MET^, except that a relatively lower level of p170^MET^ was observed in TCCSUP cells (Figure 2). Altogether, co-expression of RON and MET was a universal event in uroepithelial cells, highly suggestive of the potential clinical significance of crosstalk between RON and MET.

### Effects of RON overexpression on the biological activities of cancer cells

**Figure 4:**
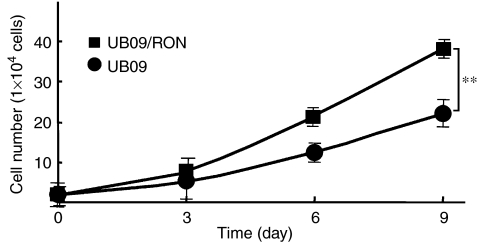
Growth curve of UB09 cells after transfection of RON. The cells (1 × 10^4^ per well for six wells) were cultured in 10% serum-containing medium with or without doxycycline (1 *μ*g ml^−1^). The cell number was calculated from 3 to 9 days using a cell counter. Those cells expressing higher RON had a significantly faster growth rate, as indicated by asterisks (*P*=0.0028, *t*-test).

As RT4 cells are resistant to transfection, UB09 cells expressing a relatively lower level of RON were transfected with plasmid DNA to express exogenous RON protein. The plasmids (80 *μ*g) pLp-TRE2-RON and pTet-Lac-Hygo at a molar ratio of 1 : 1 were transfected into UB09 cells using electroporation (180 V, 1000 *μ*F). The cells were subcultured into a six-well tray (1 × 10^4^ per well) 12 h later. The cell numbers were counted at a 3-day interval from day 0 through day 9. Overexpression of RON (UB09/RON) induced a higher growth rate of UB09 cells compared to the parental cells (*P*=0.0028) (Figure 4).

**Figure 5:**
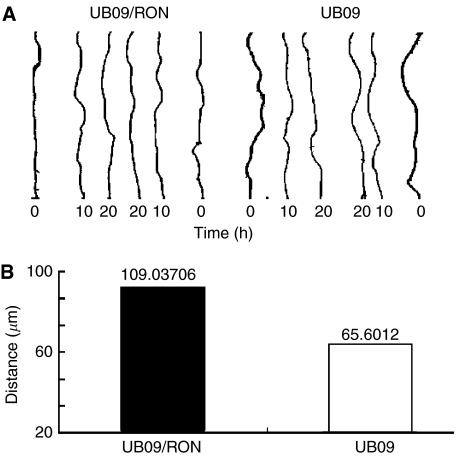
Time-lapse recording of cell migration in UB09 cells after transfection of RON. The cells (5 × 10^5^) were plated on a 1-cm cover slide and grown for 1 day, during which the cells formed a monolayer. Cells were then wounded with a yellow tip. The healing of the wounds was recorded every 20 min for a total of 20 h. (**A**) Distance of wounding of UB09/RON and UB09 cells, respectively. (**B**) Quantitative data of migration distance for UB09/RON and UB09 cells, respectively. Overexpression of RON increased the migration distance, symbolic for cell motility, of cancer cells (*P*=0.04, *t*-test).

To evaluate the effect of RON upon cell motility, UB09 cells (1 × 10^6^) were transfected and cultured on a cover glass for 24 h. Time-lapse recording was then used to evaluate the progression of wound healing produced by a yellow tip (Ho *et al*, 2005). The images were recorded every 20 min for a total of 30–48 h. It showed that UB09/RON cells migrated faster than control cells (*P*=0.04) (Figure 5).

**Figure 6:**
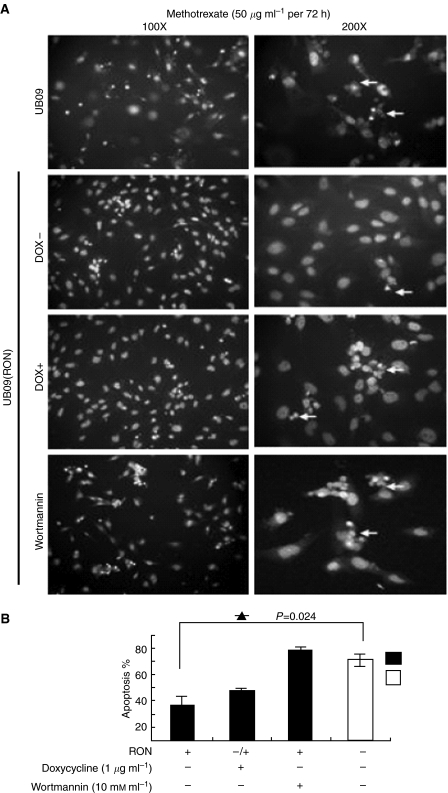
Antiapoptosis by acridine orange staining of the UB09/RON cells after MTX treatment. (**A**) UB09 cells were electroporated with 40 *μ*g of pLP-TRE2-RON and 40 *μ*g of pTet-Lac-Hyg for 12 h. Cells were first cultured in serum-containing medium for 12 h. Then that medium was replaced with medium containing only MTX (50 *μ*g ml^−1^), MTX and doxycycline (1 *μ*g ml^−1^), and, finally, wortmannin (10 nM), MTX, and doxycycline for 60 h each. All the cells were fixed with methanol for 15 min and stained with 1% acridine orange for 10 min. Cells were examined using fluorescent microscopy (left: × 100; right: × 200). Apoptotic cells are indicated by arrows. (**B**) Levels of apoptotic cells measured using apoptotic body calculation in three different fields were compared in relation to doxycycline or wortmannin treatment (*P*=0.024, *t*-test).

To test the effect of RON on apoptosis, UB09 cells were first treated with MTX for 48 h and stained with acridine orange for apoptotic cell measurement. After MTX treatment (50 *μ*g ml^−1^), 60% of the UB09 cells were apoptotic, but only 25% of the UB09/RON cells were apoptotic (*P*=0.024). The antiapoptotic effect, however, was blocked by wortmannin treatment (10 nM), a P13K inhibitor (Figure 6). Altogether, overexpression of RON increased the growth rate and motility of the cells and conferred an antiapoptotic efficacy on cancer cells through a PI3K-related signaling pathway.

### Immunohistochemical expression of RON or MET receptor in primary bladder cancer

**Figure 7:**
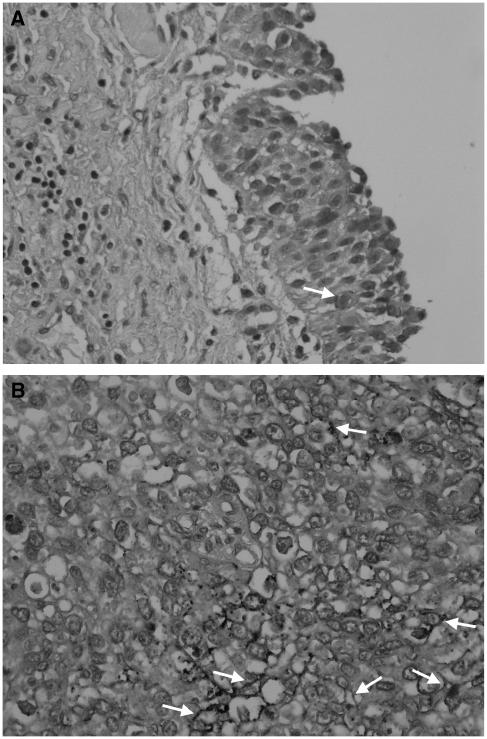
Immunohistochemical expression of RON protein in the normal urothelium and bladder cancer cells. (**A**) Membranous expression of RON receptor was seen in a few non-neoplastic uroepithelial cells (highlighted by arrow). (**B**) The arrows indicate the membranous staining of RON receptor on cancer cells, representative of a low level of RON expression (original magnification × 300).

To clarify the clinical significance of RON receptor expression, we studied the IHC expression in a transitional-cell bladder-cancer cohort (*n*=183). Membranous staining for RON was seen in only a few non-neoplastic urothelial cells (Figure 7a). Overexpression of RON was defined as membranous staining in more than 10% of tumour cells (Figure 7b). In all, 60 cases (32.8%) were classified as overexpression of RON, and 14 (23.3%) of those were high expression (summarised in Table 1). Overexpression of RON was positively associated with histological grading (*P*=0.003), larger tumours (*P*=0.003), nonpapillary contour (*P*=0.005), and tumour stage (*P*=0.01) using Fisher's exact test or the *χ*^2^ test (Table 1).

We also examined the expression of MET in this cohort (Table 1). In total, 82 cases (44.8%) overexpressed MET, and 27 (32.9%) of those were high expression. MET expression was positively associated with histological grading, nonpapillary contour, tumour size, and muscle invasion using Fisher's exact test or the *χ*^2^ test (all *P*<0.001). In all study cases, co-expression of both receptors was detected in 35 cases (19.1%), and expression of RON or MET was detected in 25 cases (13.7%), and 47 cases (25.7%), respectively (Table 2).

### Prognostic significance of co-expression of RON and MET in a bladder cancer cohort

**Figure 8:**
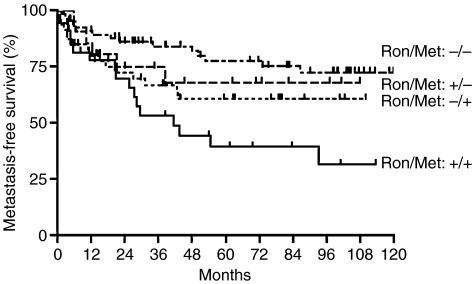
Prognostic significance of RON and/or MET expression in bladder cancer patients. Patients who co-expressed RON and MET had a significantly worse metastasis-free survival compared with patients who had single-receptor-positive tumours or no receptor expression (*P*=0.01).

In univariate analysis, none of the biological indicators analysed were correlated with tumour recurrence using the log-rank test (*P*>0.1, respectively) (data not shown), except that muscle-invasive tumours tended to have a higher risk of local recurrence (*P*=0.06). Factors associated with decreased patient survival were overexpression of RON (*P*=0.003), co-expression of RON/MET (*P*=0.005), and overexpression of MET (*P*=0.02). Patients who co-expressed RON/MET in their bladder tumours had a significantly worse overall survival rate (*P*=0.005) or metastasis-free survival rate (*P*=0.01) compared with the remaining expression status of the two receptors (Figure 8).

Multivariate analysis using a logistic regression model revealed that neither biological indicator nor expression of RON or MET or both was associated with tumour recurrence (Table 2). For long-term survival, important prognostic indicators were multiplicity (*P*=0.009), co-expression of RON/MET (*P*=0.03), expression of RON (*P*=0.04), and stage classification (*P*=0.05) using the log-rank test. Overexpression of MET by itself tended to correlate with poor patient survival in this cohort (*P*=0.06). We next used a Cox proportional hazards models to determine the RR of overall survival with 95% confidence interval (CI). The RR of poor long-term survival was 2.46 for multiple tumours, 2.22 for co-expression of RON and MET, 2.03 for RON expression alone, and 1.98 for tumour staging.

### Significance of co-expression of RON and MET in superficial bladder cancer

In the subset of superficial bladder cancer (Stages O and A), the frequency of RON and MET overexpression was 19.4% (18 out of 93) and 32.3% (30 out of 93), respectively (Table 1). Indicators associated with poor overall survival were co-expression of RON and MET (*P*=0.03) and multiplicity (*P*=0.05), with RR estimated at 3.43 (95% CI 1.16–10.12) and 2.22 (95% CI 1.01–4.91), respectively (data not shown). But expression of RON or MET or both did not predict the chance of long-term survival for patients with muscle-invasive tumours.

## DISCUSSION

In this study, we found that expression of RON or MET, or both, is positively associated with aggressive biological indicators and decreased patient survival, which supports the hypothesis that RON-related signalling events play an important role in the progression of bladder cancer. Our results are substantially in agreement with studies on primary carcinomas of the breast (Maggiora *et al*, 1998), colorectum (Zhou *et al*, 2003), liver (Chen *et al*, 1997), and ovary (Maggiora *et al*, 2003). Moreover, our *in vitro* experiments indicated that increased cell growth or motility and the antiapoptotic effect are the underlying mechanisms for RON in bladder carcinogenesis. These results are consistent with reports showing a mitogenic/invasive response induced by constitutive activation of RON (Santoro *et al*, 1996) and activation of signalling pathways, including PI3K, in regulating cell adhesion, motility, growth, and survival after stimulation with MSP (Danilkovitch *et al*, 2000; Peace *et al*, 2001).

The most intriguing observation of this investigation, however, is the prognostic importance of the co-expression of RON and MET, especially for patients with superficial bladder cancer (*P*=0.03; RR=3.43). This observation has clinical implications because this group of patients represents the largest group of urological malignancy in common practice. Co-expression of both receptors thus appears to signify a distinctive ‘invasive growth’ programme for carcinoma cells. This conclusion essentially concurs with the current hypothesis of crosstalk between subfamily members of the ErbB receptors and their prognostic significance (Wada *et al*, 1990; Sliwkowski *et al*, 1994; Pinkas-Kramarski *et al*, 1996; Chow *et al*, 2001). Therefore, evaluation of the RON and MET expression status may identify a subset of bladder cancer patients who might require more intensive treatment.

Further support for clinical relevance of RON/MET interaction in human cancer comes from *in vitro* experiments by Follenzi *et al* (2000). First, MET and RON exist on the cell membrane surface as preformed dimers before ligand stimulation, and there is a bi-directional transphosphorylation between MET and RON after stimulation with either hepatocyte growth factor or MSP. Second, although RON is a less efficient kinase than MET, formation of MET/RON complexes induces a more efficient RON transphosphorylation by MET, leading to a more sustained signal than that induced by the RON/RON homodimer. Activation of both MET and RON may thus possibly initiate a cooperative or synergistic response to their ligands. Further investigation is mandatory to clarify the benefits of combined therapies against RON/MET receptors and their signal transducers.

In our series, the most important prognostic indicators for bladder cancer patients were, first, multiple tumours at diagnosis and, second, advanced tumour stage. A similar discovery was also reported in some earlier studies (Holmang *et al*, 1995; Crew *et al*, 1997; Cheng *et al*, 2002), although no complete rationale was proposed to explain for the occurrence. Since dysplastic change in the field mucosa correlates well with clinical outcome in cancer patients, multiple tumours at diagnosis may represent a sign of unstable urothelium. Alternatively, if multiple bladder cancers are monoclonal in origin (Sidransky *et al*, 1992), the occurrence might signify an intraluminal spreading and implantation of tumour cells. A prospective study is necessary to verify the consequences of such spreading and implantation before determining what might be appropriate treatment plans for bladder cancer patients.

In summary, the results of our study indicate that RON-related molecular events are important in the progression of bladder carcinogenesis. Evaluation of the expression pattern of MET and RON is of great help in selecting bladder cancer patients for more aggressive therapy.

## Figures and Tables

**Table 1:** Distribution of RON and MET expression, and biological indicators

	**RON**			**MET**		
**Clinical characteristics**	**Positive (%)**	**Negative (%)**	***P*-value**	**Positive (%)**	**Negative (%)**	***P***-**value**
*Histological grade*						
Grade I	2 (1.1)	20 (10.9)	0.003	4 (2.2)	18 (9.8)	<0.0001
Grade II	22 (12.0)	58 (31.7)		25 (13.7)	55 (30.1)	
Grade III	36 (19.7)	45 (24.6)		83 (45.4)	28 (15.3)	
						
*Tumour shape*						
Papillary	31 (16.9)	95 (51.9)	0.005	44 (24.0)	82 (44.8)	<0.0001
Nonpapillary	29 (15.8)	28 (15.3)		38 (20.8)	19 (10.4)	
						
*Tumour size*						
⩽1 cm	5 (2.7)	31 (16.9)	0.003	8 (4.4)	28 (15.3)	0.0004
>1 to ⩽3 cm	22 (12.0)	54 (29.5)		32 (17.5)	44 (24.0)	
>3 to ⩽5 cm	20 (10.9)	22 (12.0)		28 (15.3)	14 (7.7)	
>5 cm	10 (5.5)	7 (3.8)		11 (6.0)	6 (3.3)	
N/A^a^	3 (1.6)	9 (4.9)		3 (1.6)	9 (4.9)	
						
*Multiplicity*						
Single	28 (15.3)	86 (47.0)	0.37	60 (32.8)	64 (35.0)	0.16
Multiple	22 (12.0)	37 (20.2)		22 (12.0)	37 (20.2)	
						
*Stage (TNM staging system)*						
pTa	9 (4.9)	45 (24.6)	0.01	13 (7.1)	41 (22.4)	0.001
pT1	9 (4.9)	30 (16.4)		17 (9.3)	22 (12.0)	
pT2a	16 (8.7)	17 (9.3)		23 (12.6)	10 (5.5)	
pT2b	5 (2.7)	7 (3.8)		5 (2.7)	7 (3.8)	
pT3	7 (3.8)	5 (2.7)		8 (4.4)	4 (2.2)	
pN1/pN2	8 (4.4)	11 (6.0)		13 (7.1)	6 (3.3)	
M+	2 (1.1)	2 (1.1)		2 (1.1)	2 (1.1)	
N/A^a^	4 (2.2)	6 (3.3)		1 (0.5)	9 (4.9)	

CI=confidence interval. RON=Recepteur d'Origine Nantais. MET=*c-met* protein.

a N/A: not applicable.

**Table 2:** Prognostic significance of biological indicators and expression of RON and/or MET for human bladder cancer patients (multivariate Cox regression analysis)

	**Recurrence**	**Survival**	
**Factors**	***P*-value**	***P*-value**	**RR (95% CI)**
Grade	0.60	0.41	
Stage	0.14	0.05*	1.98 (1.25–3.14)
Size	0.19	0.35	
Shape	0.85	0.60	
Multiplicity	0.88	0.009*	2.46 (1.19–3.55)
RON	0.28	0.04*	2.03 (1.08–3.53)
MET	0.40	0.06	
RON/MET	0.38	0.03*	2.22 (1.19–3.74)

CI=confidence interval. RON=Recepteur d'Origine Nantais. MET=*c-met* protein.

* *P*⩽0.05.
